# Optimal vaccination strategy against *Mycoplasma hyopneumoniae*, porcine reproductive and respiratory syndrome virus, and porcine circovirus type 2 in case of early *M. hyopneumoniae* infection

**DOI:** 10.1002/vms3.284

**Published:** 2020-05-28

**Authors:** Siyeon Yang, Taehwan Oh, Josuke Mago, Akihiro Iwakuma, Chanhee Chae

**Affiliations:** ^1^ Department of Veterinary Pathology College of Veterinary Medicine Seoul National University Seoul Republic of Korea; ^2^ Zoetis Japan Inc. Shibuya Tokyo Japan

**Keywords:** *Mycoplasma hyopneumoniae*, porcine circovirus type 2, porcine reproductive and respiratory syndrome virus, porcine respiratory disease complex

## Abstract

**Background:**

The aim of this study was to determine the optimal vaccination strategies for the control of porcine respiratory disease complex (PRDC) caused by *Mycoplasma hyopneumoniae*, porcine reproductive and respiratory syndrome virus (PRRSV), and porcine circovirus type 2 (PCV2) in case of early mycoplasmal infection.

**Methods:**

A total of 120 pigs were randomly divided into 6 groups (20 pigs per group). Four separate vaccine regimen groups were selected. Pigs from the four vaccinated groups were challenged with *M. hyopneumoniae* at 28 days old followed by a challenge of PRRSV or PCV2 at 49 days old.

**Results:**

Regardless of PRRSV or PCV2 vaccination, pigs vaccinated with one of the *M. hyopneumoniae* vaccines at 7 days old had a significantly better growth performance over the whole length of the study compared to pigs vaccinated with a second *M. hyopneumoniae* vaccine at 21 days old. Vaccination of pigs with *M. hyopneumoniae* at 7 days and PRRSV at either 7, 14 or 21 days old resulted in significantly reduced PRRSV viremia and lung lesions compared to vaccination of pigs with *M. hyopneumoniae* and PRRSV at 21 days old.

**Conclusions:**

The efficacy of the PRRSV MLV vaccine is influenced by the different timing of *M. hyopneumoniae* vaccination whereas the efficacy of the PCV2 vaccine is not. This experiment study demonstrated that early vaccination with a *M. hyopneumoniae* vaccine should be the highest priority in order to control *M. hyopneumoniae* and PRRSV infection in cases of early *M. hyopneumoniae* infection.

## Graphical abstract

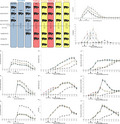

## 1 INTRODUCTION

Porcine respiratory disease complex (PRDC) is a major health concern causing devastating economic losses to the swine industry due to a decrease in growth rates, and an increase in mortality and treatment cost. PRDC is a multifactorial disease caused by a combination of infectious pathogens, environmental stressors and production system (Chae, 2016). Among the multiple aetiological agents, *Mycoplasma hyopneumoniae*, porcine reproductive and respiratory syndrome virus (PRRSV), and porcine circovirus type 2 (PCV2) are the three most common pathogens causing PRDC in Asian countries (Chae, 2016).

In the field, swine practitioners and producers use vaccination rather than antibiotic treatment and prefer to use single‐dose vaccines against *M. hyopneumoniae*, PRRSV and PCV2 in order to control PRDC efficiently. Therefore, a vaccination regimen strategy based on the natural incidence of infection is extremely important in order to avoid animals becoming infected prior to vaccination. In Japanese field conditions, pigs typically become co‐infected with PRRSV and PCV2 at around 49 days old. However, the age of mycoplasmal infection is getting younger based on early detection of *M. hyopneumoniae* DNA in laryngeal swabs collected from suckling piglets. Detection of *M. hyopneumoniae* in the tonsils and bronchus of suckling piglets is clinically significant because enzootic pneumonia caused by *M. hyopneumoniae* may start relatively early in the farrow‐to‐finish production system (Sibila et al., 2007), which is the most common production system in Japanese farms. Once weaned, these piglets are exposed to a higher mycoplasmal pressure because they come into contact with other litters of piglets increasing the risk of infection with *M. hyopneumoniae*. Vaccination against M. *hyopneumoniae* is typically administered at 21 days old. However, vaccination at 7 days of age may be necessary in order to avoid transmission of *M. hyopneumoniae* among the weaned piglets and to control enzootic pneumonia during the fattening period in farms where *M. hyopneumoniae* is detected in laryngeal swabs from suckling piglets. Although it has not been clearly shown which of these three pathogens (*M. hyopneumoniae*, PRRSV and PCV2) plays the biggest role in causing PRDC, controlling *M. hyopneumoniae* infection may be the most critical because previous studies have shown that *M. hyopneumoniae* is able to enhance the replication of PRRSV and PCV2, potentiating the severity of pneumonia in pigs (Opriessnig et al., 2004; Thacker, Halbur, Ross, Thanawongnuwech, & Thacker, 1999). Furthermore, a single‐dose vaccination against *M. hyopneumoniae* resulted in a reduction of PRRSV‐induced lung lesions whereas a single‐dose vaccination against PRRSV did not result in a reduction of *M. hyopneumoniae*‐induced lung lesions in pigs that were dually infected with PRRSV and *M. hyopneumoniae* (Park, Seo, Park, & Chae, 2014). To the best of our knowledge, no one has so far performed a comparison study where different vaccination strategies using single‐dose *M. hyopneumoniae*, PRRSV and PCV2 vaccines are compared under experimental conditions mimicking those typically observed in Japanese swine farms. The objective of this study was to compare different vaccination regimens in order to establish the optimal protection strategy against *M. hyopneumoniae*, PRRSV and PCV2 in case of early infection of *M. hyopneumoniae*.

## 2 MATERIALS AND METHODS

### 2.1 Commercial vaccines

In this study we used three monovalent vaccines; inactivated *M. hyopneumoniae* bacterin (RespiSure‐One, Zoetis, Serial No. 185372), modified‐live PRRSV vaccine (Fostera PRRS, Zoetis, Serial No. SN163540/159469, Lot No. 169588), and PCV2 subunit vaccine (Ingelvac CircoFLEX, Boehringer Ingelheim Vetmedica, Serial No. 3091076A) which were prepared and administered according to the manufacturer's instruction. A trivalent *M. hyopneumoniae*‐PCV2‐PRRSV vaccine (Ingelvac 3FLEX vaccine, Boehringer Ingelheim Vetmedica; Ingelvac CircoFLEX, Serial No. 3091076A; Ingelvac MycoFLEX, Serial No 270472A; Ingelvac PRRS MLV, Serial No. 2451124B) was prepared according to manufacturer's mixing instructions and was also used.

### 2.2 Inocula

The inocula used for challenge in this study were; Japanese PRRSV strain Jpn5‐37 (North American genotype, lineage 1) which was isolated from a 9‐week‐old diseased pig on a farm with a high mortality rate (21%) during 2007–2008 (Iseki et al., 2016), *M. hyopneumoniae* strain SNU98703 and PCV2b strain SNUVR000463 (GenBank KF871068). Japanese PRRSV strain Jpn5‐37 was kindly provided by Dr. Michihiro Takagi (Viral Research Team, National Institute of Animal Health, Tsukuba, Ibaraki, Japan). Co‐infection with PCV2 strain SNUVR000463 and *M. hyopneumoniae* strain SNU98703 was reported to cause severe pneumonia in lungs and lymphoid depletion in the lymph node of the infected pigs (Seo, Park, Park, & Chae, 2014).

### 2.3 Animals

A total of 120 colostrum‐fed, cross‐bred, conventional piglets were purchased at 7 days old from a commercial farm. Male piglets were castrated at 5 days of age. The farm was free of PRRSV and *M. hyopneumoniae* based on serological testing of the breeding herd, and long‐term clinical and slaughter history. In addition, none of the sows were immunized against PRRSV, PCV2 and *M. hyopneumoniae*. At 7 days old, piglets were seronegative for PRRSV, *M. hyopneumoniae* and PCV2. Sera samples and nasal swabs were tested by real‐time polymerase chain reaction (PCR) for PCV2 and PRRSV, and for *M. hyopneumoniae,* respectively (Dubosson et al., 2004; Gagnon et al., 2008; Wasilk et al., 2004).

### 2.4 Experimental design

**FIGURE 1:**
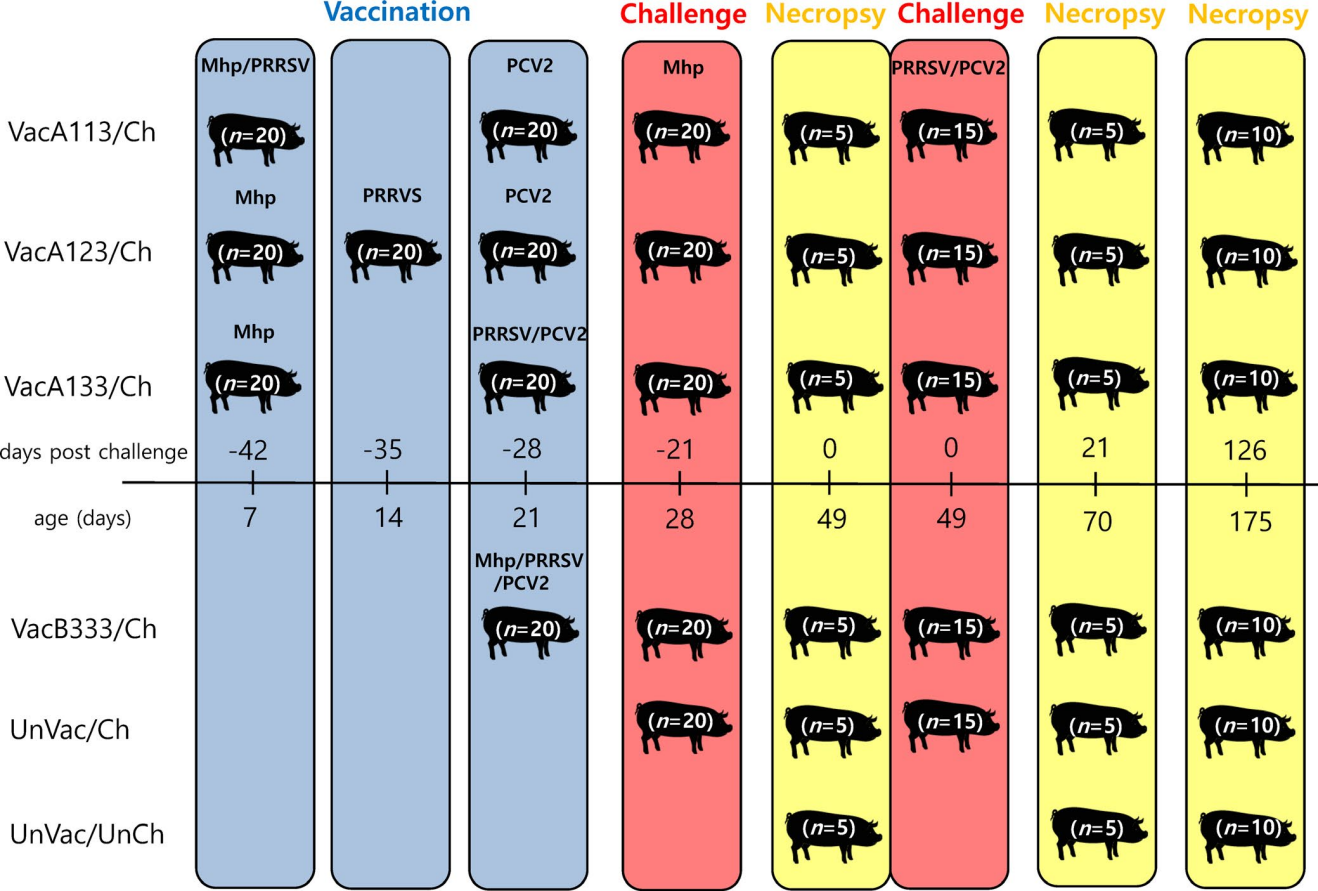
Experimental design. Pigs were administered a *M. hyopneumoniae* (Mhp), a porcine reproductive and respiratory syndrome virus (PRRSV), and a porcine circovirus type 2 (PCV2) and challenged with *M. hyopneumoniae*, PRRSV, and PCV2 on certain days as shown. A number of pigs were necropsied as shown

A total of 120 pigs were randomly divided into 6 groups using the random number generation function (Excel, Microsoft Corporation; Figure 1). Each group contained 20 piglets, 10 were male and 10 were female. All the pigs were randomly assigned into 12 rooms. Each individual room contained 10 pens with an individual pig assigned to each pen.

Pigs in the VacA113/Ch group were administered one dose of RespiSure‐One and Fostera PRRS on the left side and right side of the neck, respectively, at 7 days old, as well as a dose of CircoFLEX on the left side of the neck at 21 days old. Pigs in the VacA123/Ch group were administered one dose of RespiSure‐One on the left side of the neck at 7 days old, one dose of Fostera PRRS on the right side of the neck at 14 days old, and one dose of CircoFLEX on the left side of the neck at 21 days old. Pigs in the VacA133/Ch group were administered one dose of RespiSure‐One on the left side of the neck at 7 days old, and concurrently one dose of Fostera PRRS and CircoFLEX on the right and left side of the neck, respectively, at 21 days old. Pigs in VacB333/Ch group were administered one dose of Ingelvac 3FLEX vaccine on the right side of the neck at 21 days old. The pigs in the UnVac/Ch and UnVac/UnCh groups were administered one dose of phosphate‐buffered saline (PBS, 0.01M, pH 7.4) at 7 days old, 14 days old and 21 days old.

At −21 days post‐challenge (dpc, 28 days old), the pigs in the VacA113/Ch, VacA123/Ch, VacA133/Ch, VacB333/Ch and UnVac/Ch groups were inoculated with *M. hyopneumoniae*. Prior to inoculation, pigs were injected intramuscularly with a mixture of xylazine hydrochloride (Rompun, Bayer Korea) tiletamine hydrochloride and zolazepam hydrochloride (Zoletil 50, Virbac Korea). Seven millilitres of *M. hyopneumoniae* culture medium containing 10^7^ colour changing units (CCU)/ml were administered intratracheally as previously described (Marchioro et al., 2014; Van Reeth, Nauwynck, & Pensaert, 2000).

At 0 dpc (49 days old), pigs in the VacA113/Ch, VacA123/Ch, VacA133/Ch, VacB333/Ch and UnVac/Ch groups were challenged with PRRSV and PCV2. First, they were intranasally inoculated with 3 ml of PRRSV virus stock containing 1.2 × 10^5^ of 50% tissue culture infective dose (TCID_50_)/ml. Five hours after PRRSV inoculation, pigs were again intranasally administered 3 ml of PCV2 virus stock containing 1.2 × 10^5^ TCID_50_/ml. The 5 hr interval between challenges was applied in order to avoid mixture of two pathogens and potentially a decrease in infectivity. Pigs in the UnVac/UnCh group were the negative control.

Blood and nasal swab was collected from individual pigs at −42 (7 days old), −35, −28 (21 days old), −21, −14 (35 days old), −7, 0 (49 days old), 7, 14, 21 (70 days old), 28, 35, 42 (91 days old), 70, 98 (147 days old), and 126 (175 days old) dpc. Five pigs from each group were randomly selected for euthanasia and necropsy at 0 (49 days old) and 21 (70 days old) dpc. Ten pigs from each group were sedated by an intravenous injection of sodium pentobarbital and then euthanized by electrocution at 126 (175 days old) dpc as previously described (Beaver et al., 2001). Tissues were collected from each pig at necropsy. Tissues were fixed for 24 hr in 10% neutral buffered formalin, and embedded in paraffin.

### 2.5 Clinical observation

Pigs were monitored daily for clinical signs and scored weekly using scores ranging from 0 (normal) to 6 (severe dyspnoea and abdominal breathing; Halbur et al., 1995). Observers were blinded to vaccination and type of vaccine status. Mortality rate was calculated as the number of pigs that died divided by the number of pigs initially assigned to that group within batch. Pigs that died or were culled throughout the study were necropsied.

The live weight of each pig was measured at 7 (−42 dpc), 28 (−21 dpc), 49 (0 dpc), 70 (21 dpc), 147 (98 dpc) and 175 (126 dpc; the time of final necropsy) days of age. The average daily weight gain (ADWG; grams/pig/day) was analysed over five time periods: (a) between 7 and 28, (b) between 28 and 49, (c) between 49 and 70, (d) between 70 and 147, and (e) between 147 and 175. ADWG during the different production stages was calculated as the difference between the starting and final weight divided by the duration of the stage. Data for dead or removed pigs were included from the calculation.

### 2.6 Quantification of *M. hyopneumoniae* DNA in nasal swabs

DNA was extracted from nasal swabs using a commercial kit (QIAamp DNA Mini Kit, QIAGEN). *M. hyopneumoniae* genomic DNA copy numbers were quantified by real‐time PCR (Dubosson et al., 2004).

### 2.7 Quantification of PRRSV RNA in blood

Serum samples were collected from individual pigs and RNA was extracted and PRRSV genomic cDNA copy numbers were quantified, as previously described (Wilson et al., 2013). Real‐time PCR was also performed for the PRRSV MLV vaccine viruses and challenge PRRSV to quantify their respective genomic cDNA copies (Han, Seo, Park, & Chae, 2014; Park, Seo, Han, Kang, & Chae, 2014; Wilson et al., 2013).

### 2.8 Quantification of PCV2 DNA in blood

DNA was extracted from serum samples using a commercial DNA extraction kit (QIAamp DNA Mini Kit, QIAGEN). Real‐time PCR was used to quantify PCV2 genomic DNA copy numbers (Gagnon et al., 2008).

### 2.9 Serology

The serum samples collected from piglets were also tested for antibodies against PRRSV using IDEXX PRRS X3 Ab test (IDEXX Laboratories Inc.), for antibodies against *M. hyopneumoniae* with *M*. *hyo*. Ab test (IDEXX Laboratories Inc.), and for antibodies against PCV2 with SERELISA PCV2 Ab Mono Blocking (Synbiotics). Serum samples were considered positive for PRRSV and *M. hyopneumoniae* if the S/P ratio was ≥0.4. Sample titres of anti‐PCV2 antibodies were calculated based on a single dilution using the calculation sheet supplied by the manufacturer.

### 2.10 Enzyme‐linked immunospot assay

The numbers of *M. hyopneumoniae*‐, PRRSV‐ and PCV2‐specific interferon‐γ secreting cells (IFN‐γ‐SC) were determined in vitro by stimulating peripheral blood mononuclear cells (PBMC) using the challenge *M. hyopneumoniae* strain, PRRSV and PCV2 as previously described (Díaz & Mateu, 2005; Meier et al., 2003; Park, Seo, Han, et al., 2014; Thacker, Thacker, Kuhn, Hawkins, & Ray, 2000).

### 2.11 Interleukin‐10

The interleukin‐10 (IL‐10) protein levels in the supernatants of PBMC cultures (2 × 10^6^ cells per well, 250 μl) challenged for 20 hr with PRRSV (MOI of 0.01) or phytohemagglutinin (10 μg/ml) were quantified by using a commercial swine ELISA kit (Invitrogen) according to the manufacturer's instructions. Detection limits for IL‐10 were 3.0 pg/ml.

### 2.12 Pathology

The severity of macroscopic lung lesions was given a score in order to estimate the percentage of the lung affected by pneumonia. The scoring was done by two pathologists (authors Oh and Chae) at the Seoul National University (Seoul, Republic of Korea). For the entire lung (100 points) were assigned as follows; 10 points each to the right cranial lobe, right middle lobe, left cranial lobe and left middle lobe, 27.5 points each to the right caudal lobe and left caudal lobe, and 5 points to the accessory lobe (Halbur et al., 1995).

Lung and lymphoid tissue sections were blindly examined by two veterinary pathologists (Oh and Chae). Mycoplasmal pneumonia lesions were scored (0–6) based on the severity of peribronchiolar and perivascular lymphoid tissue hyperplasia (Thacker et al., 1999). Interstitial pneumonia lesions were scored (0–6) based on the severity of interstitial pneumonia as previously described (Halbur et al., 1995).

### 2.13 Statistical analyses

Prior to statistical analysis, real‐time PCR data were transformed to log_10_ values. Data were tested for normal distribution using Shapiro–Wilk test. One‐way analysis of variance (ANOVA) was performed for variables that showed normal distribution (ADWG, *M. hyopneumoniae* DNA, PRRSV RNA, PCV2 DNA, serology, macroscopic lung lesions and IFN‐γ‐SC). When a one‐way ANOVA showed statistical significance, a pairwise test using Tukey's adjustment was performed to determine the significance of group differences at each time point. Kruskal–Wallis test was used with variable without a normal distribution (respiratory sign and microscopic lung lesions). When the non‐parametric Kruskal–Wallis showed statistical significance, Mann–Whitney test was performed to determine the significance of group differences at each time point. A *P* value <0.05 was considered as statistical significance.

## 3 RESULTS

### 3.1 Clinical observation

**FIGURE 2:**
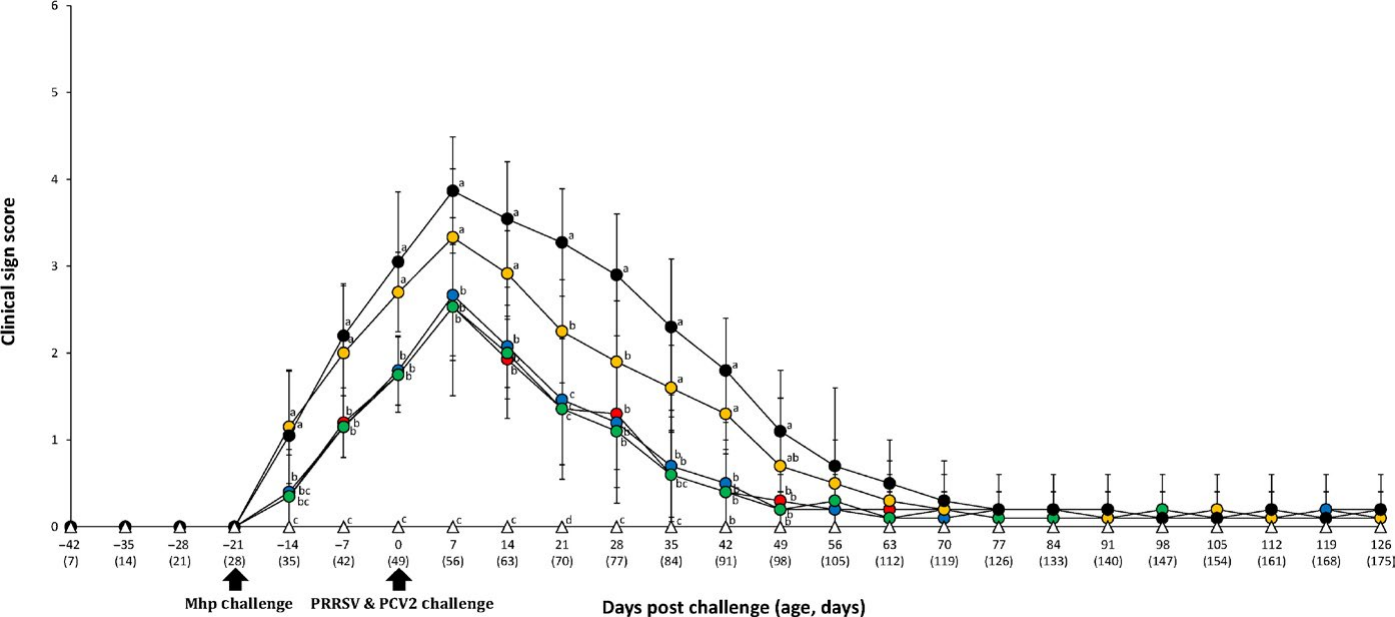
Clinical respiratory sign scores in pigs from VacA113/Ch (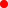), VacA123/Ch (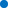), VacA133/Ch (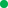), VacB333/Ch (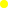), UnVac/Ch (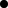) and UnVac/UnCh (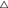) groups. Variation is expressed as the standard deviation. Different letters within a sampling point mean statistically significant differences (*p* < .05)

Clinical signs were recorded weekly and the mean respiratory scores were compared among the six different groups. Between −14 to 21 and 35 to 42 dpc, pigs from the VacA113/Ch, VacA123/Ch, VacA133/Ch and UnVac/UnCh groups had significantly lower (*p* < .05) mean respiratory scores compared to the VacB333/Ch and UnVac/Ch groups. At 21 dpc, pigs from the VacB333/Ch group had significantly lower (*p* < .05) mean respiratory scores compared to the UnVac/Ch group. At 28 dpc, pigs from the VacA113/Ch, VacA123/Ch, VacA133/Ch, VacB333/Ch and UnVac/UnCh groups had significantly lower (*p* < .05) mean respiratory scores compared to the UnVac/Ch group. At 49 dpc, pigs from the VacA113/Ch, VacA123/Ch, VacA133/Ch and UnVac/UnCh groups had significantly (*p* < .05) lower mean respiratory scores compared to the UnVac/Ch group (Figure 2).

Pigs from the VacA113/Ch, VacA123/Ch and VacA133/Ch groups had significantly higher (*p* < .05) mean respiratory scores compared to the UnVac/UnCh group from −7 to 21 dpc. Pigs from VacA123/Ch group had significantly higher (*p* < .05) mean respiratory scores compared to the UnVac/UnCh group at −14 dpc. Pigs from the VacA123/Ch and VacA133/Ch groups had significantly higher (*p* < .05) mean respiratory scores compared to the UnVac/UnCh group at 35 dpc. No clinical respiratory signs were observed in the pigs from the UnVac/UnCh group (Figure 2).

A total of 11 pigs died during the whole study period. Death correlated with severe pneumonia in 9 of the 11 pigs: 1 at 12 dpc in the VacA123/Ch group; 1 at 13 dpc in the VacA133/Ch group; 3 at 9, 10 and 12 dpc in the VacB333/Ch group; and 4 at 8, 11, 12 and 13 dpc in the UnVac/Ch group. One pig in the VacA123/Ch group had positive for PRRSV and PCV2 in lung and lymphoid tissue by real‐time PCR. One pig in the VacA133/Ch group had positive for PCV2 in lung and lymphoid tissue by real‐time PCR. Three pigs in VacB333/Ch group and four pigs in the UnVac/Ch group had positive for *M. hyopneumoniae*, PRRSV and PCV2 in lung and for PRRSV and PCV2 in lymphoid tissue by real‐time PCR. Two pigs (1 at 8 dpc in VacA113/Ch and 1 at 12 dpc in VacA123/Ch groups) died due to unknown reasons without histopathological lesions. These two pigs had negative for *M. hyopneumoniae*, PRRSV and PCV2 in lung and lymphoid tissue by real‐time PCR.

### 3.2 Growth performance

No statistical difference was observed in the average body weight (±standard deviation) among the six groups at 7 days old which was the start of the experiment. The average body weight for each group at 7 days old was, for the VacA113/Ch group (2.63 Kg ± 0.29), VacA123/Ch group (2.63 Kg ± 0.31), VacA133/Ch group (2.63 Kg ± 0.39), VacB333/Ch group (2.64 Kg ± 0.34), UnVac/Ch group (2.66 Kg ± 0.31) and UnVac/UnCh group (2.67 Kg ± 0.33). There was no significant difference in ADWG among the six groups between 7 and 49 days old. Pigs from the VacA113/Ch, VacA123/Ch, VacA133/Ch and UnVac/UnCh groups had significantly higher (*p* < .05) ADWG compared to the VacB333/Ch and UnVac/Ch groups between 49 and 70 days old. Pigs from the VacB333/Ch group had significantly higher (*p* < .05) ADWG compared to the UnVac/Ch group between 49 and 70 days old. Pigs from the VacA113/Ch, VacA123/Ch, VacA133/Ch, VacB333/Ch and UnVac/UnCh groups had significantly higher (*p* < .05) ADWG compared to the UnVac/Ch group between 70 and 147 days old. There was no significant difference in ADWG among the 6 groups between 147 and 175 days old. The overall ADWG (from 7 to 175 days old) in pigs from the VacA113/Ch, VacA123/Ch, VacA133/Ch and UnVac/UnCh groups was significantly higher (*p* < .05) compared to the VacB333/Ch and UnVac/Ch groups. The overall ADWG (from 7 to 175 days old) in pigs from the VacB333/Ch group was significantly higher (*p* < .05) compared to the UnVac/Ch (Table 1).

**TABLE 1:** Average daily weight gain (ADWG, mean ± standard deviation) in pigs from different groups

Period between days post‐challenge	ADWG (grams/day/pig)*						
	Age (days)	VacA113/Ch	VacA123/Ch	VacA133/Ch	VacB333/Ch	UnVac/Ch	UnVac/UnCh
−42 to −21	7 to 28	239.85 ± 34.66	243.65 ± 29.28	245.11 ± 33.86	232.21 ± 36.14	236.61 ± 26.71	235.95 ± 42.60
−21 to 0	28 to 49	363.91 ± 62.86	364.29 ± 77.85	366.67 ± 60.61	347.62 ± 54.07	355.06 ± 49.08	380.95 ± 58.52
0 to 21	49 to 70	521.77 ± 50.42^a^	519.41 ± 52.61^a^	519.39 ± 65.72^a^	449.60 ± 53.15^b^	326.84 ± 32.51^c^	579.37 ± 60.35^a^
21 to 98	70 to 147	733.25 ± 28.83^a^	727.53 ± 31.64^a^	732.21 ± 24.43^a^	702.86 ± 16.67^a^	659.48 ± 23.92^b^	726.88 ± 32.72^a^
98 to 126	147 to 175	742.86 ± 52.49	739.29 ± 48.05	746.43 ± 77.34	750.00 ± 61.86	714.29 ± 73.19	750.00 ± 91.75
−42 to 126	7 to 175	598.33 ± 12.99^a^	598.04 ± 13.64^a^	598.69 ± 13.49^a^	576.13 ± 12.25^b^	537.56 ± 16.51^c^	607.08 ± 15.18^a^

Note

Different superscript letters^a,b,c^ within a sampling point mean statistically significant differences (*p* < .05).

* The live weight of each pig was measured at 7 (−42 days post‐challenge; dpc), 28 (−21 dpc), 49 (0 dpc), 70 (21 dpc), 147 (98 dpc) and 175 (126 dpc; the time of final necropsy) days of age among 6 groups.

### 3.3 Quantification of *M. hyopneumoniae* DNA in nasal swabs

**FIGURE 3:**
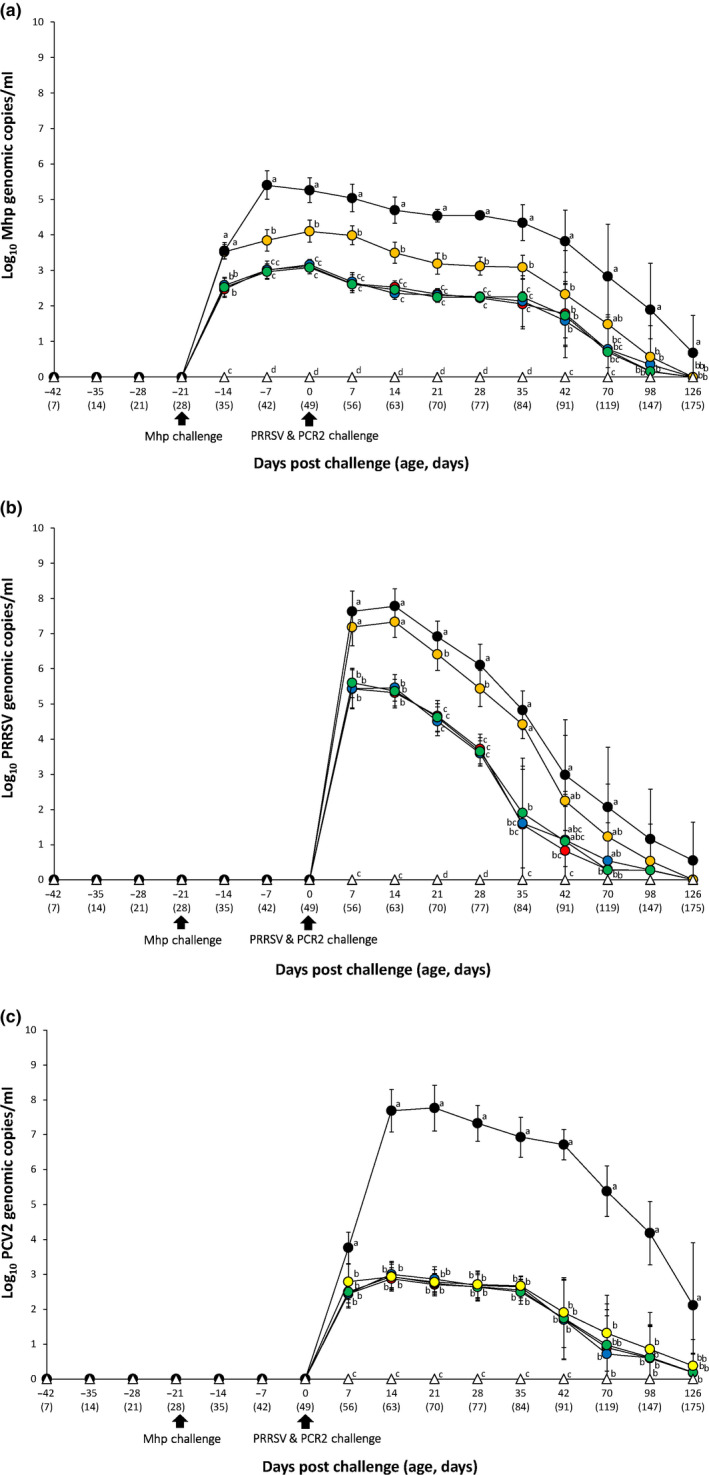
Quantification of *M. hyopneumoniae* DNA, PRRSV RNA and PCV2 DNA. Mean values of the genomic copy numbers of *M. hyopneumoniae* DNA in the nasal swabs (A), the genomic copy numbers of PRRS RNA in the serum samples (B), and the genomic copy numbers of PCV2 DNA in the serum samples (C) in the different groups: VacA113/Ch (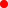), VacA123/Ch (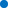), VacA133/Ch (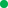), VacB333/Ch (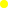), UnVac/Ch (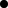) and UnVac/UnCh (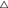) groups. Variation is expressed as the standard deviation. Different letters within a sampling point mean statistically significant differences (*p* < .05)

At the time of challenge, no genomic copies of *M. hyopneumoniae* were detected in any of the nasal samples from any of the six groups. Pigs from the VacB333/Ch and UnVac/Ch groups had a significantly higher (*p* < .05) number of genomic copies of *M. hyopneumoniae* in their nasal swabs compared to the VacA113/Ch, VacA123/Ch and VacA133/Ch groups between −14 and 35 dpc. Pigs from the UnVac/Ch group had a significantly higher (*p* < .05) number of genomic copies of *M. hyopneumoniae* in their nasal swabs compared to the VacB333/Ch group between −7 to 42 and 98 to 126 dpc. Pigs from the UnVac/Ch group had a significantly higher (*p* < .05) number of genomic copies of *M. hyopneumoniae* in their nasal swabs compared to the VacA113/Ch, VacA123/Ch and VacA133/Ch groups between 42 to 126 dpc. No genomic copies of *M. hyopneumoniae* were detected in any of the nasal samples collected from the UnVac/UnCh group throughout the study (Figure 3a).

### 3.4 Quantification of PRRSV RNA in blood

Serum samples from pigs in all six groups were tested for the presence of PRRSV genomic copies. No genomic copies of PRRSV were detected in any of the pigs prior to challenge. Pigs from the VacA113/Ch, VacA123/Ch and VacA133/Ch groups experienced a significant (*p* < .05) reduction in PRRSV genomic copies in their blood compared to the VacB333/Ch and UnVac/Ch groups between 7 and 35 dpc. Pigs from the VacB333/Ch group had a significantly (*p* < .05) reduced number of genomic copies of PRRSV in their blood compared to the UnVac/Ch group between 21 and 28 dpc. Pigs from the VacA113/Ch group had a significantly (*p* < .05) reduced number of genomic copies of PRRSV in their blood compared to the UnVac/Ch group at 42 and 70 dpc. Pigs from the VacA133/Ch group had a significantly (*p* < .05) reduced number of genomic copies of PRRSV in their blood compared to the UnVac/Ch group at 70 dpc. No genomic copies of PRRSV were detected in pigs from the UnVac/UnCh group throughout the experiment (Figure 3b).

### 3.5 Quantification of PCV2 DNA in blood

Serum samples were also tested for the presence of PCV2. Prior to challenge, all serum samples were negative for PCV2. Pigs from the VacA113/Ch, VacA123/Ch, VacA133/Ch and VacB333/Ch groups had a significantly (*p* < .05) reduced number of PCV2 genomic copies compared to the UnVac/Ch group between 7 and 126 dpc. No genomic copies of PCV2 were detected in pigs from the UnVac/UnCh group throughout the experiment (Figure 3c).

### 3.6 Immunological responses against *M. hyopneumoniae*

**FIGURE 4:**
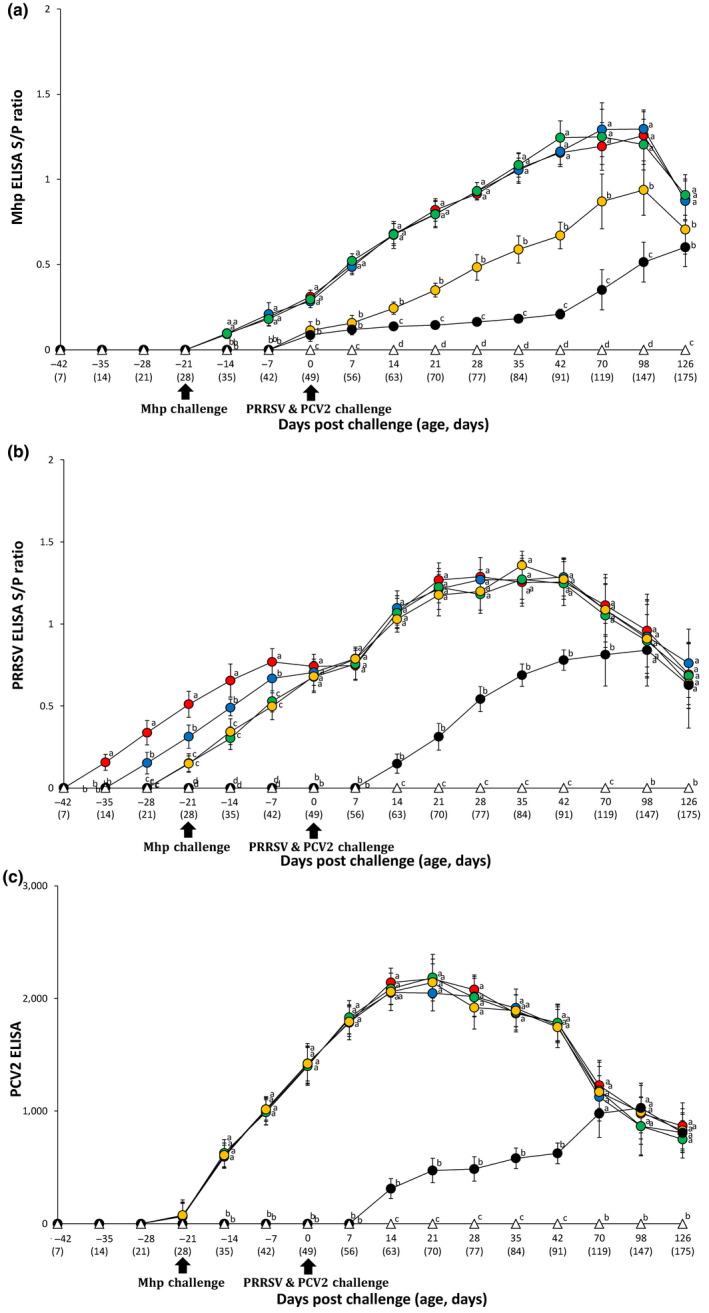
Enzyme‐linked immunosorbent assay. Mean values of the *M. hyopneumoniae*‐specific ELISA S/P ratio (A), the PRRSV‐specific ELISA S/P ratio (B), and the PCV2‐specific ELISA antibody levels (C) in the different groups: VacA113/Ch (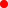), VacA123/Ch (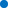), VacA133/Ch (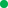), VacB333/Ch (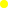), UnVac/Ch (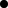) and UnVac/UnCh (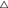) groups. Variation is expressed as the standard deviation. Different letters within a sampling point mean statistically significant differences (*p* < .05)

Pigs from the VacA113/Ch, VacA123/Ch and VacA133/Ch groups had significantly higher (*p* < .05) *M. hyopneumoniae*‐specific ELISA S/P ratio compared to the VacB333/Ch and UnVac/Ch groups between −14 and 126 dpc. Pigs from the VacB333/Ch group had significantly higher (*p* < .05) *M. hyopneumoniae*‐specific ELISA S/P ratio compared to the UnVac/Ch group between 14 and 98 dpc (Figure 4a).

**FIGURE 5:**
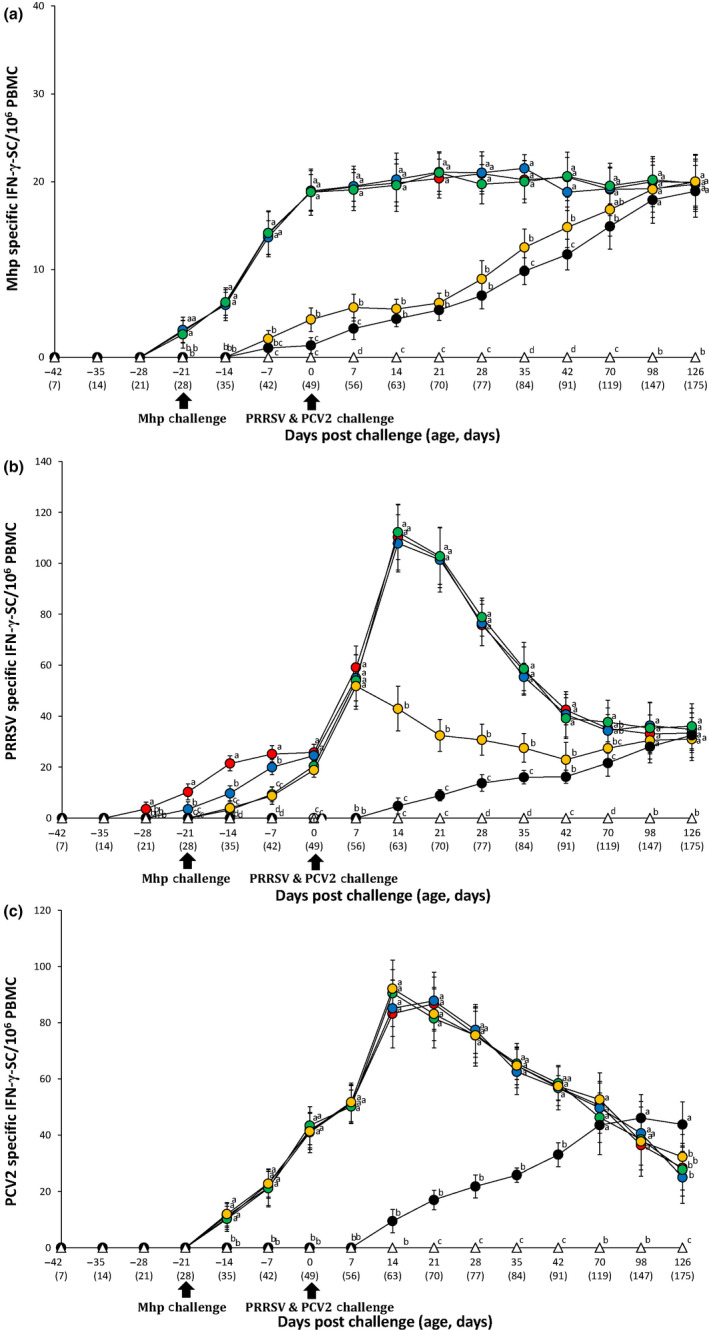
Frequency of interferon‐γ secreting cells (IFN‐γ‐SC). Mean number of *M*. *hyopneumoniae*‐specific IFN‐γ‐SC (A), PRRSV‐specific IFN‐γ‐SC (B) and PCV2‐specific IFN‐γ‐SC (C) in the different groups: VacA113/Ch (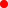), VacA123/Ch (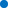), VacA133/Ch (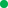), VacB333/Ch (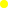), UnVac/Ch (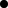) and UnVac/UnCh (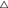) groups. Variation is expressed as the standard deviation. Different letters within a sampling point mean statistically significant differences (*p* < .05)

T‐cell response was determined by quantifying *M. hyopneumoniae*‐specific IFN‐γ‐SC. Pigs from the VacA113/Ch, VacA123/Ch and VacA133/Ch groups had significantly higher (*p* < .05) *M. hyopneumoniae*‐specific IFN‐γ‐SC compared to the VacB333/Ch and UnVac/Ch groups between −21 and 42 dpc. Pigs from the VacB333/Ch group had significantly higher (*p* < .05) *M. hyopneumoniae*‐specific IFN‐γ‐SC compared to the UnVac/Ch group at 0, 7, 35 and 42 dpc. Pigs from the VacA113/Ch, VacA123/Ch and VacA133/Ch groups had significantly higher (*p* < .05) *M. hyopneumoniae*‐specific IFN‐γ‐SC compared to the UnVac/Ch group at 70 dpc. No anti‐*M. hyopneumoniae* antibodies or *M. hyopneumoniae*‐specific IFN‐γ‐SC were detected in pigs from the UnVac/UnCh group (Figure 5a).

### 3.7 Immunological responses against PRRSV

PRRSV‐specific antibody response and the number of PRRSV‐specific IFN‐γ‐SC were also examined. Pigs from the VacA113/Ch group had significantly higher (*p* < .05) PRRSV‐specific ELISA S/P ratio compared to the VacA123/Ch, VacA133/Ch, VacB333/Ch and UnVac/Ch groups between −35 and −7 dpc. Pigs from the VacA123/Ch group had significantly higher (*p* < .05) PRRSV‐specific ELISA S/P ratio compared to the VacA133/Ch, VacB333/Ch and UnVac/Ch groups between −28 and −7 dpc. Pigs from the VacA133/Ch and VacB333/Ch groups had significantly (*p* < .05) higher PRRSV‐specific ELISA S/P ratio compared to the UnVac/Ch group between −21 and −7 dpc. Pigs from the VacA113/Ch, VacA123/Ch, VacA133/Ch and VacB333/Ch groups had significantly higher (*p* < .05) PRRSV‐specific ELISA S/P ratio compared to the UnVac/Ch groups between 0 and 70 dpc (Figure 4b).

Pigs from the VacA113/Ch group had significantly higher (*p* < .05) PRRSV‐specific IFN‐γ‐SC compared to the VacA123/Ch, VacA133/Ch, VacB333/Ch and UnVac/Ch groups between −28 and −7 dpc. Pigs from the VacA123/Ch group had significantly higher (*p* < .05) PRRSV‐specific IFN‐γ‐SC compared to the VacA133/Ch, VacB333/Ch and UnVac/Ch groups between −21 and −7 dpc. Pigs from the VacA133/Ch and VacB333/Ch groups had significantly higher (*p* < .05) PRRSV‐specific IFN‐γ‐SC compared to the UnVac/Ch group at −14 and −7 dpc. Pigs from the VacA113/Ch, VacA123/Ch, VacA133/Ch and VacB333/Ch groups had significantly higher (*p* < .05) PRRSV‐specific IFN‐γ‐SC compared to the UnVac/Ch group between 0 and 35 dpc. Pigs from the VacA113/Ch, VacA123/Ch and VacA133/Ch groups had significantly higher (*p* < .05) PRRSV‐specific IFN‐γ‐SC compared to the VacB333/Ch group between 14 and 42 dpc. Pigs from the VacA113/Ch, VacA123/Ch and VacA133/Ch groups had significantly higher (*p* < .05) PRRSV‐specific IFN‐γ‐SC compared to the UnVac/Ch group at 42 and 70 dpc. Pigs from the VacA133/Ch group had significantly higher (*p* < .05) PRRSV‐specific IFN‐γ‐SC compared to VacB333/Ch group at 70 dpc. No anti‐PRRSV antibodies or PRRSV‐specific IFN‐γ‐SC were detected in pigs from the UnVac/UnCh group (Figure 5b).

### 3.8 Immunological responses against PCV2

Pigs from the VacA113/Ch, VacA123/Ch, VacA133/Ch and VacB333/Ch groups had significantly higher (*p* < .05) PCV2 antibody titres compared to the UnVac/Ch group between −14 and 42 dpc (Figure 4c). Pigs from the VacA113/Ch, VacA123/Ch, VacA133/Ch and VacB333/Ch groups had significantly higher (*p* < .05) PCV2‐specific IFN‐γ‐SC compared to the UnVac/Ch group between −14 and 42 dpc. Pigs from the UnVac/Ch group had significantly higher (*p* < .05) PCV2‐specific IFN‐γ‐SC compared to the VacA113/Ch, VacA123/Ch, VacA133/Ch and VacB333/Ch groups at 126 dpc. No anti‐PCV2 antibodies or PCV2‐specific IFN‐γ‐SC were detected in pigs from the UnVac/UnCh group (Figure 5c).

### 3.9 Interleukin‐10

**FIGURE 6:**
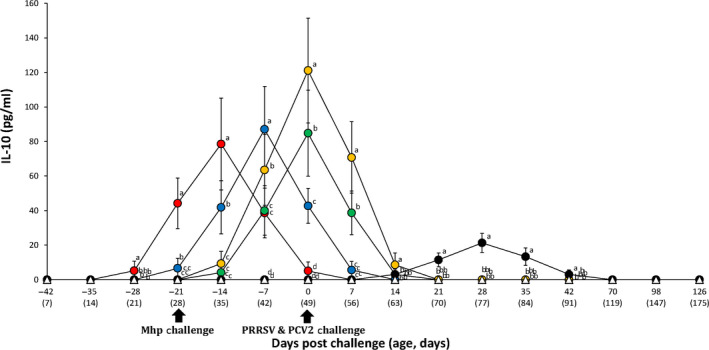
Mean values of interleukin‐10 in the serum samples in the different groups: VacA113/Ch (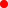), VacA123/Ch (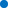), VacA133/Ch (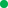), VacB333/Ch (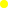), UnVac/Ch (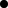) and UnVac/UnCh (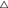) groups. Variation is expressed as the standard deviation. Different letters within a sampling point mean statistically significant differences (*p* < .05)

Pigs from the VacA113/Ch group had significantly higher (*p* < .05) IL‐10 levels compared to the VacA123/Ch, VacA133/Ch, VacB333/Ch, and UnVac/Ch groups between −28 and −14 dpc. Pigs from the VacA123/Ch group had significantly higher (*p* < .05) IL‐10 levels compared to the VacA133/Ch, VacB333/Ch and UnVac/Ch groups between −21 and −14 dpc. Pigs from the VacA123/Ch group had significantly higher (*p* < .05) IL‐10 levels compared to the VacA113/Ch, VacA133/Ch, VacB333/Ch and UnVac/Ch groups at −7 dpc. Pigs from the VacB333/Ch group had significantly higher (*p* < .05) IL‐10 levels compared to the VacA113/Ch, VacA133/Ch and UnVac/Ch groups at −7 dpc. Pigs from the VacA113/Ch and VacA133/Ch groups had significantly higher (*p* < .05) IL‐10 levels compared to the UnVac/Ch group at −7 dpc. Pigs from the VacB333/Ch group had significantly higher (*p* < .05) IL‐10 levels compared to the VacA113/Ch, VacA123/Ch, VacA133/Ch and UnVac/Ch groups between 0 and 14 dpc. Pigs from the VacA133/Ch group had significantly higher (*p* < .05) IL‐10 levels compared to the VacA113/Ch, VacA123/Ch and UnVac/Ch groups between 0 and 7 dpc. Pigs from the VacA123/Ch group had significantly higher (*p* < .05) IL‐10 levels compared to the VacA113/Ch and UnVac/Ch groups at 0 dpc. Pigs from the UnVac/Ch group had significantly higher (*p* < .05) IL‐10 levels compared to the VacA113/Ch, VacA123/Ch, VacA133/Ch and VacB333/Ch groups between 21 and 42 dpc. No IL‐10 levels were detected in pigs from the UnVac/UnCh group (Figure 6).

### 3.10 Pathology

Pigs from the VacA113/Ch, VacA123/Ch and VacA133/Ch groups had significantly lower (*p* < .05) macroscopic lung lesion scores compared to the UnVac/Ch group at 0, 21 and 126 dpc. Pigs from the VacA113/Ch, VacA123/Ch and VacA133/Ch groups had significantly lower (*p* < .05) macroscopic lung lesion scores compared to the VacB333/Ch group at 0 and 21 dpc. Pigs from the VacB333/Ch group had significantly lower (*p* < .05) macroscopic lung lesion scores compared to the UnVac/Ch group at 21 and 126 dpc (Table 2).

**TABLE 2:** Macroscopic and microscopic lung lesions scores (mean ± standard deviation) in pigs from different groups at 126 days post‐challenge (175 days of age)

Pathology	Groups					
	VacA113/Ch	VacA123/Ch	VacA133/Ch	VacB333/Ch	UnVac/Ch	UnVac/UnCh
Macroscopic lung lesions	2.00 ± 1.48^b^	2.10 ± 1.30^b^	2.20 ± 1.47^b^	3.65 ± 2.56^b^	16.85 ± 6.16^a^	0.00 ± 0.00^b^
Microscopic lung lesions						
Mycoplasmal pneumonia	0.12 ± 0.13^b^	0.10 ± 0.18^b^	0.12 ± 0.16^b^	0.18 ± 0.21^b^	0.52 ± 0.26^a^	0.00 ± 0.00^b^
Interstitial pneumonia	0.12 ± 0.13^b^	0.16 ± 0.17^b^	0.12 ± 0.16^b^	0.20 ± 0.22^b^	0.66 ± 0.28^a^	0.00 ± 0.00^b^

Note

Different superscript letters^a,b^ mean statistically significant differences (*p* < .05).

Pigs from the VacA113/Ch, VacA123/Ch and VacA133/Ch groups had significantly lower (*p* < .05) microscopic mycoplasmal lung lesion scores compared to the VacB333/Ch and UnVac/Ch groups at 0 and 21 dpc. Pigs from the VacA113/Ch, VacA123/Ch, VacA133/Ch and VacB333/Ch groups had significantly lower (*p* < .05) microscopic mycoplasmal lung lesion scores compared to the UnVac/Ch group at 126 dpc. Pigs from the VacA113/Ch, VacA123/Ch and VacA133/Ch groups had significantly lower (*p* < .05) microscopic interstitial lung lesion scores compared to the VacB333/Ch and UnVac/Ch groups at 21 dpc. Pigs from the VacA113/Ch, VacA123/Ch, VacA133/Ch and VacB333/Ch groups had significantly lower (*p* < .05) microscopic interstitial lung lesion scores compared to the UnVac/Ch group at 126 dpc (Table 2).

## 4 DISCUSSION

In this study we have shown that vaccination of pigs with a *M. hyopneumoniae*, PRRSV and PCV2 vaccine can be effective in protecting against these pathogens in cases of early *M. hyopneumoniae* challenge. In particular, *M. hyopneumoniae* vaccination at 7 days old proved to be the most effective (VacA113/Ch, VacA123/Ch and VacA133/Ch groups) since it resulted in the best growth performance compared to the group vaccinated with *M. hyopneumoniae* at 21 days old (VacB333/Ch group) regardless of PRRSV and PCV2 vaccination. These results emphasize the need for optimal vaccination strategies depending on infection patterns in the field. The PRDC model used in this study was designed based on recent infection patterns in Japanese field conditions. Under these conditions, *M. hyopneumoniae* infection is occurring at an earlier age, suggesting that the *M. hyopneumoniae* vaccine should be administered at a younger age than the current vaccination at 3 weeks old. However, unlike this study, piglets may have the maternally derived antibodies under common Japanese field conditions. An experimental challenge study showed that vaccination of pigs at 7 days old with the same *M. hyopneumoniae* vaccine used in this study, was effective in reducing lung lesions even in the presence of maternally derived antibodies at a titre considerably higher than what is typically seen in the field (Wilson et al., 2012, 2013). Therefore, efficacy of *M. hyopneumoniae* vaccines is not interfered with maternally derived antibodies. These results indicate that the timing of vaccination against *M. hyopneumoniae* should be determined based on the age of infection rather than the presence of maternally derived antibodies. In addition, the timing of PCV2 challenge mimics natural infection in Japanese field conditions. In general, PCV2 infection starts at the end of the nursery or at the beginning of fattening periods, however, is usually quite variable depending on the farm (Takahaki, Toki, Nishiyama, Morimatsu, & Murakami, 2010). Hence, vaccinated pigs were challenged with PCV2 at 49 days of age based on infection patterns in the field. However, in this study, the timing of PCV2 challenge is likely to be younger than the time of PCV2 infection occurred in some Japanese farms.

*M. hyopneumoniae* vaccination at 7 days old resulted in higher reduction of lung lesions after challenge at 28 days old, compared to mycoplasma vaccination at 21 days old. A concern is that early vaccination may not be effective due to a lack of immune system maturity within the piglets resulting in decreased vaccine efficacy. In study regarding to early *M. hyopneumoniae* vaccination, vaccination of pigs with *M. hyopneumoniae* at 7 days old has been shown to induce a stronger cell‐mediated immune response compared to vaccination at 21 days old (Seo et al., 2013) suggesting a protective advantage of early vaccination. In addition, cell‐mediated immunity as measured by activation of IFN‐γ‐SC plays an important role in protective immune response against *M. hyopneumoniae* (Thacker, Thacker, Kuhn, et al., 2000). Therefore, the pig immune system is unique in many ways (Butler, 2009; Sinkora et al., 2003) which attributed to the piglet's ability to develop protective immunity from early vaccination.

Our results should be interpreted carefully. Pigs in the VacA133/Ch, VacA123/Ch and VacA133/Ch groups were vaccinated at 7 days old while pigs in the VacB333/Ch groups were vaccinated at 21 days old, according to manufacturer's claim followed by *M*. *hyopneumoniae* challenge at 28 days old. In general, *M. hyopneumoniae* vaccines generally take at least 14 days to induce effective immunity (Seo et al., 2013). The pigs in the VacA133/Ch, VacA123/Ch and VacA133/Ch groups have enough time (21 days) to mount immunity before *M. hyopneumoniae* challenge, while the pigs in the VacB333/Ch group had only 7 days to the *M. hyopneumoniae* challenge. As a result, pigs in the VacB333/Ch group did not have enough time to induce a protective immunity to control *M*. *hyopneumoniae* infection. These differences other than vaccine schedule may be responsible for lower vaccine efficacies against *M. hyopneumoniae* and subsequent PRRSV and PCV2 challenges among pigs in the VacB333/Ch group as compared with those in other vaccinated groups. Further study is needed to evaluate the effect of 7 days vaccination of *M. hyopneumoniae* vaccine used in the VacB333/Ch group.

The efficacy of the PRRSV MLV vaccine is influenced by the different timing of *M*. *hyopneumoniae* vaccination. Interestingly, the PRRSV vaccination at 21 days old in the VacA133/Ch group was able to reduce PRRSV viremia significantly compared to the PRRSV vaccination at 21 days old in the VacB333/Ch group. The difference between the two groups is that they use different *M*. *hyopneumoniae* bacterins and PRRSV MLV vaccines, and the time of *M*. *hyopneumoniae* vaccination. Pigs in the VacA133/Ch and VacB333/Ch groups were vaccinated at 7 and 21 days old, respectively, according to manufacturer's claim followed by *M*. *hyopneumoniae* challenge at 28 days old. Therefore, pigs in the VacB333/Ch group did not have enough time to mount an immune response against *M*. *hyopneumoniae*. Previous studies have also shown that a challenge with *M*. *hyopneumoniae* could potentiate the replication of a subsequent PRRSV challenge exacerbating pneumonia caused by PRRSV (Thacker et al., 1999; Thacker, Thacker, Young, & Halbur, 2000; Alstine, Stevenson, & Kanitz, 1996). In addition, it has also been reported that *M*. *hyopneumoniae* infection can impair the efficacy of a PRRSV MLV vaccine (Thanawongnuwech & Thacker, 2003). Taken together, these results suggest that the timing of the *M*. *hyopneumoniae* challenge affects the efficacy of the PRRSV MLV vaccine and that administration and efficacy of the PRRSV MLV vaccine depends on the administration time and efficacy of the *M*. *hyopneumoniae* vaccine. Alternatively, the difference on the reduction of PRRSV viremia between VacA133/Ch and VacB333/Ch groups may be due to differences on the PRRSV MLV vaccine viruses. Two PRRSV MLV vaccine viruses share only 91.3% nucleotide identity for open reading frame (ORF) 5. In addition, the Japanese PRRSV strain Jpn5‐37 used in this study has similar homology to the two vaccine viruses (85.5% and 84% for Fostera PRRS and for Ingelvac PRRS MLV, respectively) based on ORF5 amino acids sequences, although the full genome was not analysed. This eliminates genetic similarity as an advantage of one vaccine over the other. These results may suggest that a Fostera PRRS vaccine virus in the VacA133/Ch group and challenge the Japanese PRRSV strain Jpn5‐37 may be closely related antigenically. Nevertheless, the two PRRSV MLV vaccines could not be compared accurately because of different administration time of *M. hyopneumoniae* vaccination and use of *M. hyopneumoniae* bacterins. Therefore, further studies are needed to compare two PRRSV MLV vaccines directly under the same experimental conditions.

PRRSV infection induces production of IL‐10 which has been shown to be immunosuppressive through the induction of regulatory Tcells (Conti et al., 2003; Flores‐Mendoza, Silva‐Campa, Reséndiz, Osorio, & Hernández, 2008; Suradhat, Thanawongnuwech, & Poovorawan, 2003). However, there are conflicting opinions whether this is a survival mechanism utilized by PRRSV (Rodríguez‐Gómez et al., 2015; Silva‐Campa et al., 2010). Because IL‐10 is a known potent immunosuppressive cytokine, induction of IL‐10 following PRRSV vaccination may cause interference of the PCV2 vaccine's efficacy. In our study, induction of PCV2‐specific IFN‐γ‐SC was concurrent with the increase of IL‐10 production, suggesting limited interference by IL‐10 with the immune responses induced by the PCV2 vaccine. However, further studies are needed to confirm limited interference under the same vaccination and non‐challenging experimental conditions.

In contrast to PRRSV MLV vaccine, the efficacy of the PCV2 vaccine is not influenced by the different timing of *M. hyopneumoniae* vaccination. In this study, there were no significant differences in the reduction of PCV2 viremia between *M. hyopneumoniae* vaccination at 7 days old (VacA113/Ch, VacA123/Ch and VacA133/Ch groups) and 21 days old (VacB333/Ch group). Despite the fact that *M. hyopneumoniae* enhances the replication of PCV2 and potentiates lymphoid lesions caused by PCV2 (Opriessnig et al., 2004), the relationship between the *M. hyopneumoniae* and PCV2 vaccines is somewhat independent (Seo et al., 2014) *M. hyopneumoniae* infection did not impair the induction of humoral and cell‐mediated immunity by the PCV2 vaccine and vice versa (Seo et al., 2014). Therefore, the fact that there is only 1 week interval between *M. hyopneumoniae* vaccination and challenge does not affect the ability of the PCV2 vaccine to reduce PCV2 viremia.

It is important to note that, in the present study, each single‐dose vaccine of *M. hyopenumoniae* and PRRSV was administered at 7 days of age (VacA113/Ch group) into separate anatomical sites. It is possible that concurrent vaccination may interfere with immune responses to each vaccine. However, the immune responses elicited in this study by the concurrent administering of the *M. hyopneumoniae* and PRRSV vaccines do not appear to be significantly different from immune responses following single administering of each vaccine based on a previous study (Jeong et al., 2018). Therefore, we believe that concurrent vaccination conducted in this study does not interfere with immune responses from either vaccine.

Economic benefit is one of the most important parameters for the evaluation of vaccine. In general, economic benefit is estimated on market weight. Protection of *M. hyopneumoniae* infection by the early *M. hyopneumoniae* vaccination helped increase the market weight by 3.7 kg/pig; 103.1 kg in early *M. hyopneumoniae* vaccinated (VacA113/Ch, VacA123/Ch, and (VacA133/Ch) groups and 99.4 kilograms in late *M. hyopneumoniae* vaccinated (VacB333/Ch) group. Improved market weight of 3.7 kg/pig increased revenue by 8.9 US$ (exchange rate; US $1.00 = 1,193 Korean Won) per pig.

In conclusion, *M. hyopneumoniae* infection not only affects the efficacy of PRRSV MLV vaccine, it also directly affects growth performance. The results obtained from the experimental conditions above may not be accurately reflected in the field conditions. Therefore, swine practitioners and producers should modify the vaccine strategy according to field situation. In case of early *M. hyopneumoniae* infection, early vaccination against *M*. *hyopneumoniae* should be the first priority in order to control PRDC efficiently. In addition, maternally derived antibodies, timing of infection, management or housing differences, and production style (i.e. continuous, all‐in‐all‐out and multi‐site system) can influence the vaccination strategy. This experimental study provides an optimal vaccination strategy suitable for the control of PRDC caused by *M. hyopneumoniae* and PRRSV in case of early *M*. *hyopneumoniae* infection in pig farms.

## CONFLICT OF INTEREST

Drs. Josuke Mago and Akihiro Iwakuma are employees of Zoetis Japan Inc. The authors declare no conflict of interest with respect to their authorship or the publication of this article.

## AUTHOR'S CONTRIBUTION

SY was involved in performance of the experimental trials, data analysis and writing of the manuscript. TO was involved in preparation of the inoculum and laboratory analysis. JM and AI were involved in data analysis and writing of the manuscript. CC was involved in development of protocol, design of the study, review of the final manuscript, approval for publication. All authors read and approved the final manuscript.

## ETHICAL STATEMENT

All of the methods were previously approved by the Seoul National University Institutional Animal Care and Use, and Ethics Committee. Sample collection was carried out according to the animal welfare code of Korea.
